# Associations between Parenting Style and Mental Health in Children and Adolescents Aged 11–17 Years: Results of the KiGGS Cohort Study (Second Follow-Up)

**DOI:** 10.3390/children8080672

**Published:** 2021-08-02

**Authors:** Özge Azman, Elvira Mauz, Matthias Reitzle, Raimund Geene, Heike Hölling, Petra Rattay

**Affiliations:** 1 Department Epidemiology and Health Monitoring, Robert Koch Institute, Nordufer 20, 13353 Berlin, Germany; MauzE@rki.de (E.M.); HoellingH@rki.de (H.H.); RattayP@rki.de (P.R.); 2 Department of Developmental Psychology, Friedrich Schiller University of Jena, Am Steiger 3/1, 07743 Jena, Germany; Matthias.Reitzle@uni-jena.de; 3 Department of Health & Education, Berlin School of Public Health, Alice Salomon University of Applied Sciences, Alice-Salomon-Platz 5, 12627 Berlin, Germany; raimund.geene@charite.de

**Keywords:** family, parenting, mental health, strengths and difficulties questionnaire, childhood, adolescence, Germany, socioeconomic position, migration

## Abstract

Few studies from Germany have investigated the associations between parenting style and children’s and adolescents’ health. Little attention has been directed to whether these associations vary with familial socioeconomic or migration status. The aim of this analysis was to investigate the association between parenting style and the mental health of children and adolescents aged 11–17 years using data from the KiGGS cohort study (second follow-up). We calculated mean Strengths and Difficulties Questionnaire (SDQ) total difficulties scores stratified by parenting style (authoritative, permissive, demanding–controlling, emotional distancing). Linear regression analyses adjusted for age, gender, socioeconomic status, migration status, and family status were performed. We also analyzed moderating effects of socioeconomic and migrations status on associations between parenting style and SDQ scores. There were only small differences between the permissive and the authoritative parenting styles. Significantly higher mean scores were observed for the demanding–controlling and emotional distancing styles for both the mother and father. These associations persisted after full adjustment and did not vary by socioeconomic or migration status. Parenting behavior is an important predictor of children’s and adolescents’ mental health. The promotion of good relationships within families and improving parenting skills offer promising approaches for health promotion in young people.

## 1. Introduction

Family represents a central form of socialization that shapes the health development of children and adolescents [1]. Everyday family interactions in which children and adolescents learn basic knowledge, skills, and attitudes have an essential influence on their health and social, physical, and psychological development [2]. Several family factors, including attachment to a parent, emotional support, family cohesion, parents’ psychosocial stress, and parenting styles have been discussed in the international literature as important predictors of psychosocial development in childhood and adolescence [3,4]. Parenting behaviors have a lasting impact on child development, with certain parenting behaviors being risk factors for a child’s mental health [5,6]. Psychological and emotional behavioral disorders in childhood and adolescence can impact the quality of life of affected individuals and can last into adulthood [7]. In Germany, the prevalence of mental health problems in children and adolescents remains high [7]. Therefore, the identification of protective factors is an important consideration for public health, especially in developing prevention and health promotion concepts. In detail, the present study aims to analyze the association between mothers’ and fathers’ parenting styles and the mental health of children and adolescents aged 11–17 years, also considering also the mediating and moderating effects of social determinants (e.g., SES, migration status, and family structure).

### 1.1. State of Research

#### 1.1.1. Parenting Style Research

Parenting styles represent parents’ consistent attitude toward their children, and are based on certain attitudes and patterns of behavior. This includes various interactions and techniques with which parents try to shape their children’s long-term development [8]. The work of Baumrind [9,10] forms the basic building block for research on parenting styles. Based on observations of parent–child relationships, Baumrind examined associations between parenting practices and social behavior and personality development in children. This resulted in the development of a parenting typology that distinguished authoritarian, permissive, and authoritative parenting styles [11]. Later, Maccoby and Martin [12] extended this parenting typology to include a fourth parenting pattern, which was the rejecting-neglectful parenting style [13,14].

The multidimensional approach of Baumrind’s parenting typology offered the possibility of considering complex mechanisms of parental upbringing and provided a popular basis for further research on parenting styles [15,16]. Parenting dimensions were further differentiated in the development of the theoretical concept for assessing parenting styles.

In a study conducted in German-speaking areas, Reitzle et al. [6] extended the parenting styles based on Baumrind’s conceptualization, including through analyses of interdimensional interactions using the Zurich Brief Questionnaire for the Assessment of Parental Behaviors (ZKE). That study suggested the authoritative parenting style was characterized by a high level of warmth/support and demands/control, which resulted in clear and recognizable rules of behavior for children, along with below-average values for psychological pressure. The demanding–controlling parenting style showed low values in the emotional dimension but maintained a high level of control and psychological pressure. This style corresponded to the authoritarian parenting style discussed in the literature. A permissive parenting style was characterized by slightly above-average values for warmth/support and low levels of rules/control and psychological pressure. This indicated that these parents offered their child moderate levels of emotional support but set a low value on adherence to limits and rules [6,13]. Reitzle et al. [6] described another parenting style, namely emotional distancing. This parenting style was characterized by below-average scores on all three dimensions. Parents with the emotional distancing parenting style neither offered their child emotional support nor expect compliance with rules [6]. 

A German language analysis was conducted in 1994 in which a total of 877 students aged 11–17 years from Zurich were questioned about the parenting style of their parents [6]. That study showed the authoritative parenting style was the most common configuration among both mothers and fathers. The second most common parenting style was the permissive style, followed by emotional distancing, with the demanding–controlling parenting style being least common [6]. 

A Swiss study [17] investigated children with a migration background and showed that compared with Swiss adolescents, migrant children perceived higher psychological control and rejection by both parents. Children with a migration background perceived their mothers as less supportive and felt a low level of emotional maternal warmth [17].

#### 1.1.2. Mental Health of Children and Adolescents

The prevalence of mental health problems among children and adolescents in Germany has been relatively stable in recent decades. The German Health Interview and Examination Survey for Children and Adolescents (KiGGS) baseline survey (2003–2006) as well as the KiGGS Wave 1 study (2009–2012) that used the Strengths and Difficulties Questionnaire (SDQ) reported that 20% of the participating children in Germany were “psychologically conspicuous.” In the KiGGS Wave 2 study (2014–2017), the rate of children with mental health problems was 16.9%, with the prevalence among boys being significantly higher than among girls (19.1% vs. 14.5%). The difference between boys and girls was particularly marked in the group aged 3–14 years, whereas the prevalence of emotional and behavioral problems in boys and girls aged 15–17 years was similar [7]. However, boys were more frequently affected by externalizing problems, whereas girls more frequently had internalizing, emotional problems [18].

The development of mental health problems in childhood and adolescence is affected by various risk factors. An adverse social situation in a family can cause psychosocial stress, which can lead to harsh parenting practices and a non-conducive family climate; in turn, this can have long-term adverse effects on children and adolescents [19]. Children and adolescents from families with a low socioeconomic status (SES) are more frequently affected by mental health problems than children and adolescents from socioeconomically better-off families [7]. Furthermore, several studies observed a higher prevalence of mental health problems in adolescents with a migration background. Children and adolescents with a two-sided migration background more often reported mental health problems than children and adolescents with a one-sided or no migration background [20]. Family structure can be another determinant of health in children. For example, emotional and behavioral problems are more prevalent in children from stepfamilies and single-parent families than in children who live together with both biological parents in the same household [21].

#### 1.1.3. Associations between Parenting Style and Mental Health among Children and Adolescents

Several studies have reported an association between an authoritative parenting style and positive developmental effects in children and adolescents. Authoritatively raised children more often appear to be independent, self-confident, and emotionally stable [5,14,22]. They also show a better performance, higher social skills, and more active coping strategies in school than children reared with other parenting styles [23,24,25]. 

Kuppens and Ceulemans [26] reported that children raised in an authoritarian manner had the least favorable scores on all SDQ subscales, particularly with reference to the authoritative parenting style. Other studies suggested that a permissive parenting style resulted in high SDQ total difficulties scores compared with the authoritarian parenting style [27], and reported negative effects associated with permissively raised elementary school children, such as high levels of aggressiveness, antisocial behavior problems, and lack of self-discipline [28,29,30]. 

The ZKE validation study conducted in German-speaking areas found that authoritative parenting was associated with positive developmental outcomes in children [6]. Both maternal and paternal support were associated with positive effects and may therefore protect children and adolescents from internalizing symptoms (e.g., depressive feelings) or externalizing symptoms (e.g., problematic substance use) [6,31]. Furthermore, positive developmental effects for permissively raised adolescents have also been reported [6,25]. 

Conversely, authoritarian and emotional distancing parenting styles were associated with negative developmental outcomes, such as higher symptom distress, low self-esteem, and the development of avoidant coping strategies [6]. A high level of psychological pressure in the authoritarian parenting style can lead to internalizing symptoms, whereas a low level of rules/control can lead to externalizing behavioral problems [6,32]. For example, a study that used the ZKE with a German sample of 274 students (aged 14–17 years) found that students whose parents had an authoritarian parenting style had higher depersonalization scores, lower preference for coping strategies, and higher anxiety compared with students who were raised authoritatively or permissively [25].

Parenting styles can have different effects on children and adolescents depending on their cultural background. Although predominantly positive effects are seen for the authoritative parenting style in Western cultures, this cannot necessarily be applied to all cultures [33,34]. Furthermore, adolescents from ethnic minorities showed lower positive developmental outcomes associated with the authoritative parenting style [35].

### 1.2. Aim of This Study and Research Questions

As noted above, few studies in German-speaking areas have investigated associations between parenting style and the mental health of children and adolescents. Therefore, this study aimed to examine associations between different parenting styles and the mental health of children and adolescents aged 11–17 years.

There are strong associations between social determinants (e.g., SES, migration status, and family structure) and mental health in children and adolescents, and the parenting styles of mothers and fathers may be considered along with these social determinants. Therefore, we included these social determinants as possible control and moderator variables. Given the paucity of studies on differences in the associations between parenting style and the mental health of children and adolescents in the context of family SES and migration status, this analysis intended to help close this research gap. 

Specifically, the following research questions were analyzed:

1. Do the parenting styles of mothers and fathers differ by the children’s and adolescents’ gender and age, SES, migration status, and family status?
2. Are there associations between the parenting style of mothers and fathers and their children’s and adolescents’ mental health?
3. Do the associations between mother’s and father’s parenting styles and children’s and adolescents’ mental health persist when controlled for SES, migration status, and family status?
4. Do the associations between children’s and adolescents’ mental health and mother’s and father’s parenting styles differ by SES or migration status?

## 2. Materials and Methods

### 2.1. Data

This analysis was based on data drawn from the second follow-up of the KiGGS cohort study. This is the longitudinal component of the KiGGS study, which was conducted by the Robert Koch Institute as part of nationwide health monitoring. Data for the second follow-up were collected from 2014 to 2017 using a combined health interview and examination survey. All 17,641 participants who participated in the KiGGS baseline study (2003–2006) were invited to complete the second follow-up. All individuals who continued to reside in the former study location were asked to participate in the health interview and examination survey in the second follow-up. Individuals who had moved away from the area were only invited to participate in the health interview survey. Overall, 61.5% of participants from the baseline study participated in Wave 2 [36]. This sample included 4596 children and adolescents aged 11–17 years. For more information about the second follow-up of the KiGGS cohort study, see Lange et al. [36].

### 2.2. Variables

The outcome variable was emotional and behavioral problems, which were assessed in the KiGGS Wave 2 using the parent version of the SDQ for children and adolescents aged 10–17 years [37]. The SDQ is a validated, reliable, and internationally approved screening instrument [38,39]. The SDQ symptom questionnaire comprises four problem scales: Conduct problems, hyperactivity/inattention problems, emotional problems, and peer relationships problems. Each subscale has five items that are answered by parents on a three-point Likert scale (0 = not true, 1 = somewhat true, 2 = certainly true). The 20 items across the four problem subscales are summed to give an SDQ total difficulties score (range 0–40) [40]. We used the metric variable to consider the full information content of the SDQ total difficulties score in the analyses.

The predictor variables were the parenting styles of the mother and father, which were assessed separately using the German version of the Zurich Brief Questionnaire for the Assessment of Parental Behaviors (D-ZKE; formerly known as the ZKE) [6,41]. The D-ZKE for mothers and fathers was used for the first time in Wave 2 of the KiGGS cohort study and was completed by children and adolescents aged 10–17 years [37]. The instrument has 27 items on three dimensions: Warmth/support, rules/control, and psychological pressure. Example items are: “My mother/father praises me when I do something well” (warmth/support), “My mother/father has clear rules and regulations on how I should behave“ (rules/control), and “My mother/father demands that I perform better at school than others” (psychological pressure). Each item is answered on a four-point scale from 0 “not true” to 3 “completely true.” The questionnaire reliably and validly measures the three mentioned dimensions [6]. Cluster analysis consistent with the procedure reported in Reitzle et al. [6] was used to form parenting styles based on the interaction of the three dimensions. These parenting styles were categorized as: Authoritative, demanding-controlling, emotional distancing, and permissive [41]. The parenting styles identified in previous studies were almost perfectly replicated in the KiGGS data [42]. 

The mediator variables were SES, migration status, and family status. SES was determined by an index of the parents’ educational status, occupational status, and income and differentiated into three categories: Low, medium, and high [43]. The child’s migration status was differentiated as none, a one-sided, or a two-sided migration background [44]. The family status was determined by whether the child lived in a shared household with both biological parents. SES and migration status were also used as moderator variables. The control variables were the child’s age and gender, mother’s status (biological mother vs. stepmother) and father’s status (biological father vs. stepfather). 

### 2.3. Statistical Analysis

Because the D-ZKE was used for the first time in KiGGS Wave 2, we only conducted cross-sectional analyses. In the first step, the parenting styles of mothers and fathers were described overall as well as stratified by the children’s gender and age, SES, migration status, and family status. 

In the second step, the means and 95% confidence intervals (CI) for the SDQ total difficulties score were calculated to be stratified by mother’s and father’s parenting styles. This was performed separately for girls and boys. 

In the third step, multiple linear regression analyses were performed using the SDQ total difficulties score as the metric dependent variable and parenting styles of mothers and fathers as the predictor variables. The authoritative parenting style was used as the reference category (dummy coding comparing each group with the reference group). A basic model (Model 1) was calculated by including age, gender, and mother’s status versus father’s status as the control variables. Next, a fully adjusted model (Model 2) was developed, including the above control variables as well as the mediator variables (SES, migration status, and family status). Mediation effects were only analyzed by changes in means and *p*-values; no further mediation analysis was conducted in which the total effect was split into direct and indirect effects.

**Figure 1:**
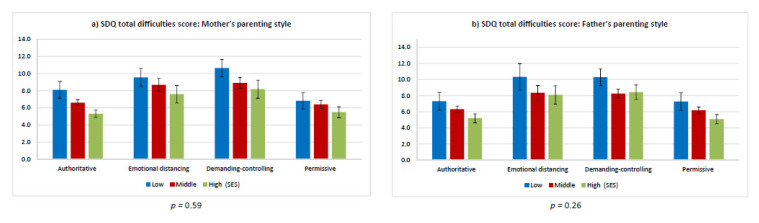
Predicted mean Strengths and Difficulties Questionnaire total difficulties scores stratified by parenting style and socioeconomic status (with 95% confidence intervals and *p*-values (joint Wald test)). (**a**) SDQ total difficulties scores: Mother’s parenting style. (**b**) SDQ total difficulties scores: Father’s parenting style.

**Figure 2:**
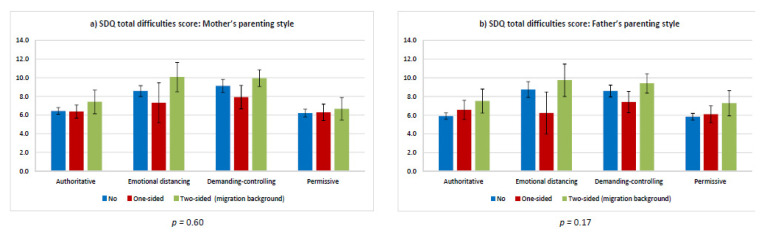
Predicted mean Strengths and Difficulties Questionnaire total difficulties scores stratified by parenting style and migration background (with 95% confidence intervals and *p*-values (joint Wald test)). (**a**) SDQ total difficulties scores: Mother’s parenting style. (**b**) SDQ total difficulties scores: Father’s parenting style.

In the fourth step, the moderator effects of SES and migration status were analyzed by integrating interactions terms for SES and parenting style (Model 3) as well as migration status and parenting style (Model 4) into the linear regressions. To test group differences in the association between parenting styles and SDQ total difficulties score we performed joint Wald tests for the interaction terms of parenting style and SES, and for parenting style and migration status. Predictive margins for the SDQ total difficulties score were calculated stratified by parenting style and SES and migration background, respectively, and graphically presented in Figure 1 and Figure 2.

As all linear regression analyses showed no significant differences between girls and boys, no stratified results were presented, but gender was included as a control variable. Means, β-coefficients, and predictive margins were calculated with 95% CI as effect measures. Statistical significance was set at *p*-values less than 0.05. All analyses were performed with weighted variables using the survey procedure with STATA (Version SE 15.1). 

The KiGGS baseline study provided representative data on the health of children and adolescents in Germany because of the random selection of participants and subsequent weighting of the data. However, this representativeness was not guaranteed in the second follow-up. Willingness to participate in the second follow-up varied by sociodemographic characteristics (e.g., age, education), which created some attrition bias. Therefore, a weighting factor was calculated that considered the cross-sectional weight of the KiGGS baseline data multiplied by the longitudinal weight that compensated for possible attrition bias because of differential dropout. The dropout weight was represented by the inverse probability of participation in Wave 2. This resulted in a higher weighting for groups that tended to be less willing to participate in the follow-up [36,45], and re-established the representative nature of the KiGGS baseline sample.

Only children and adolescents aged 11–17 years were included in these analyses. We excluded individuals with missing information on parenting style (maternal parenting style: n = 313; paternal parenting style: n = 595). In addition, children and adolescents who answered the questions on parenting style but did not live in a shared household with the assessed parent were excluded (maternal parenting style: n = 182; paternal parenting style: n = 510), as it could not be ruled out that children assessed the parenting style of a parent differently if that parent did not live in the same household. The sample used for the present analyses comprised 4101 children and adolescents who reported the parenting style of their mother or stepmother and lived in the same household, and 3491 children and adolescents who reported the parenting style of their father or stepfather and lived in the same household. The description of the study sample is presented in Table 1.

## 3. Results

The authoritative parenting style was the most frequent configuration for the overall sample for both mothers and fathers (Table 2 and Table 3). Maternal and paternal parenting styles showed significant differences in frequency by age group, SES, and migration status. Maternal parenting was less often characterized by the authoritative style in boys and girls aged 14–17 years compared with those aged 11–13 years. However, the permissive style and emotional distancing were more frequent in the older age group (14–17 years). For fathers, the frequency of the demanding–controlling parenting style remained the same in both age groups. Compared with the younger age group, the permissive style was more prevalent for boys and emotional distancing was more prevalent for girls and boys in the older age group (Table 3). Girls and boys from families with a high SES were mostly raised authoritatively or permissively by both their mother and father. In contrast, 40.5% of boys in the low SES group described the parenting style of their father as demanding-controlling. In addition, in families with a two-sided migration background, the parenting style of the father was described as demanding–controlling by 45.6% of the boys, whereas this applied only to 22.1% of boys without a migration background (Table 3). A permissive parenting style of fathers was more common among boys without a migration background and least common among boys with a two-sided migration background. These differences were also observed in girls. A demanding–controlling parenting style for mothers was most prevalent among boys and girls with two-sided migration background (Table 2).

Table 4 presents the mean SDQ total difficulties scores stratified by the parenting style of the mother and father. Permissively raised boys showed the lowest mean score, followed by authoritatively raised boys. For girls, there were no significant differences in means between permissive and authoritative parenting. In comparison, girls and boys whose mothers and fathers practiced demanding–controlling or emotional distancing parenting styles had the highest mean total difficulties scores.

Table 5 presents the results of the linear regression analysis for the SDQ total difficulties score. When controlled for children’s age and gender, and mother’s status or father’s status Table 5, Model 1, there were no significant differences in the total difficulties score between the permissive and authoritative parenting styles for either mothers or for fathers.

For the demanding–controlling and emotional distancing parenting styles, we observed significantly higher coefficients for the SDQ total difficulties score compared with the authoritative parenting style. This was the case for both mothers and fathers. 

The strong statistically significant association between the demanding-controlling parenting style and the high SDQ total difficulties score remained even after full adjustment for SES, migration status, and family status (Table 5, Model 2). This was also the case for the emotional distancing parenting style. Therefore, SES, migration status, and family status did not mediate the effect of the mother’s or father’s parenting style on mental health problems among children and adolescents.

Figure 1 and Figure 2 show the results for the moderating effects of SES and migration status on the association between parenting styles and the SDQ total difficulties score in children and adolescents. No significant moderation effect was found for SES, but there were significant main effects for SES and parenting style. Therefore, children and adolescents in the low SES group had the highest total difficulties scores among all subgroups (Figure 1a,b). The total difficulties scores for all SES groups were higher for the emotional distancing and demanding–controlling parenting styles of both mothers and fathers than for the authoritative and permissive parenting styles. However, children with a low SES and a mother whose parenting was characterized by a permissive style had a better SDQ total difficulties score than children with a high or middle SES whose mother’s parenting style was characterized by emotional distancing or demanding–controlling.

Figure 2 presents the moderation effect of migration status on the association of parenting styles and the SDQ total difficulties score. As with SES, there was no statistically significant moderation effect by migration status. Comparison within the parenting style subgroups for mothers and fathers showed that the total difficulty score tended to be higher (non-significant) for children and adolescents with a two-sided migration background than for children and adolescents with a one-sided or no migration background (Figure 2a,b). Among children and adolescents with a two-sided migration background, a significantly lower mean was found for the permissive maternal parenting style than for the emotional distancing or demanding-controlling parenting styles (Figure 2a). A similar pattern was seen for the paternal parenting style, with overlapping CIs (Figure 2b). The scores for children with a migration background and a mother with a permissive parenting style were lower than the scores for children with no migration background whose mother’s parenting style was characterized by emotional distancing or demanding–controlling. For children and adolescents with a one-sided migration background, no significant differences in total difficulty scores were seen between the different parenting styles for either the mother or father (Figure 2a,b). Among children without a migration background, the maternal and paternal authoritative and permissive parenting styles showed significantly lower mean scores than the emotional distancing and demanding-controlling parenting styles (Figure 2a,b).

## 4. Discussion

The present study aimed to analyze the associations between mothers’ and fathers’ parenting styles (i.e., authoritative, demanding-controlling, emotional distancing, and permissive) and the mental health of children and adolescents aged 11–17 years. 

Clear differences in the parenting styles were apparent depending on the age of the child, SES, and migration status. Children aged 11–13 years were more often raised authoritatively by their parents, whereas adolescents aged 14–17 years were more often raised permissively. The present study also showed that fathers and mothers were more often emotionally distant and withdrawn in bringing up their older children compared with their younger children. Therefore, the age of the child had a major impact on mothers’ and fathers’ parenting behavior and suggested that parents’ control and rules significantly decreased during adolescence. While the authoritative and permissive parenting styles were most common in families with high SES, the prevalence of the demanding–controlling and emotionally distant parenting styles were significantly higher in families with low SES. These findings supported the results of Bergmann et al. [19] who indicated that low family social status was often associated with less supportive parenting behaviors. A similar pattern was found for migration background. Thus, the demanding–controlling parenting style for fathers was twice as common among boys and girls with a two-sided migration background compared with children without a migration background. Steinhausen et al. [17] also found that children with a migrant background experienced greater psychological control by their parents This may be because more controlling parenting behavior was consistent with the cultural beliefs and values of a family.

When considering mental health, we found that the SDQ total difficulties scores for children and adolescents varied with the parenting style of the mother and father. Consistent with existing literature, an authoritative parenting style of mothers and fathers was associated with low SDQ total difficulties scores in children and adolescents. Furthermore, the permissive parenting style was also associated with low SDQ total difficulties scores in our analysis. Other international studies found associations between a permissive parenting style with externalizing problems [27,28,29,30,46]. However, these results relate to younger children (aged 3–13 years), while positive psychological development in the context of permissive parenting styles was found in older children and adolescents aged eleven years and older [6,25], analogous to our study. It can be assumed that with the permissive parenting style, younger children more often react negatively to low levels of rules and control by parents, whereas adolescents more often react positively to the opportunity to develop their own personality without strong parental control. However, the different findings regarding the permissive parenting style indicated that further research is needed in this area. In the present study, a demanding-controlling parenting style of both mothers and fathers was associated with the highest SDQ total difficulties scores. This result was consistent with the findings of Kuppens and Ceulemans [26], who reported that having authoritarian parents was correlated with high emotional and behavioral problem scores in children. A demanding-controlling parenting style is likely to hinder the development of autonomy in adolescence and may therefore contribute to the development of mental health problems. In our study, significantly higher SDQ total difficulties scores were also observed for emotionally distant mothers or fathers. Reitzle et al. [6] found that the emotionally-distant parenting style had unfavorable developmental effects, such as a lack of active coping strategies in children and adolescents. 

In contrast to our study, several previous studies considered different parenting dimensions rather than parenting styles. For example, Barber et al. [32] showed that a high level of psychological pressure, which is characteristic of the demanding-controlling parenting style, was associated with emotional and behavioral problems in children and adolescents. Low levels of rules and control was also observed to correspond with psychological problems [32]. Furthermore, several studies found that parents’ emotional warmth and support were protective factors for children’s mental health [5,6,15]. The associations between the demanding–controlling and emotionally distant parenting style and high SDQ total difficulties scores found in our study may be attributed to a low level of parental warmth and support as well as a high level of psychological control. However, it has been shown in the literature that parenting dimensions do not occur in isolation from each other in daily life. Instead, a combination of these dimensions appears to impact a child’s development [6,14,26]. For example, based on the SDQ scores in our analyses, low levels of rules and control combined with high or moderate levels of warmth and support (permissive parenting style) were associated with psychologically favorable scores in adolescents, whereas the combination of low levels of rules and control with low levels of warmth and support from parents (emotionally distant parenting style) tended to be associated with higher total difficulties scores. Therefore, this approach to studying parenting styles had the advantage of considering specific combinations of parenting dimensions. 

With regard to the developmental task of becoming independent from parents in adolescence [47], a relaxation of demanding-controlling parenting and rules combined with a continuously good emotional parent-child relationship appears to support mental health in this developmental stage. This also corresponds to the innate psychological needs of autonomy, relatedness, and competence (as postulated in the self-determination theory), which when satisfied, enhance mental health. In contrast, excessive control, non-optimal challenges, and lack of connectedness lead to distress and mental health problems [48].

Furthermore, we examined whether the associations between parenting styles and children’s and adolescents’ mental health could be explained by different mediator variables. However, our results revealed that the effect of maternal and paternal parenting styles on mental health was not mediated by SES, migration status, or family status. Our findings showed that irrespective of the social situation of the children, the parenting styles of the mother and father are important influencing factors and a central resource for healthy mental development in adolescence.

The present study also aimed to determine whether the association between parenting style and the mental health of children and adolescents varied with SES or migration status. To our knowledge, there has been no similar study to date. Therefore, we investigated these potential moderators for the first time and revealed that the association between parenting styles and SDQ total difficulties score did not vary by SES or migration status. Instead, we found that the SDQ total difficulties score tended to be higher for children with two-sided migration backgrounds in all parenting styles. Therefore, children with a migration background may experience higher psychological stress than children without a migration background [20]. However, like children and adolescents aged 11–17 years without a migration background, they benefit from a warm and less controlling parenting style.

Our moderation analysis revealed that adolescents with a low SES and a mother with a permissive parenting style had significantly fewer mental health problems than adolescents with a high or middle SES and a mother with an emotionally distant or demanding-controlling parenting style. Therefore, the parenting behavior of the mother may somewhat cushion the negative effects of social disadvantage on adolescents’ mental health. We believe that this finding may inform strategies to support the prevention of mental health problems among young people with social disadvantage. 

The present study had some strengths and limitations. A major strength was that parenting styles were assessed from the perspective of children and adolescents, whereas children’s/adolescents’ mental health was assessed from the perspective of parents. Considering both perspectives reduced potential bias and increased the validity of our results. However, it is possible that permissive parents perceived and assessed their children’s behavior differently from parents who raised their child in a more demanding–controlling way. In addition, it is possible that the assessment of parenting style by the children and adolescents was completed in presence of their parents. Therefore, it remains unclear to what extent socially desirable response behavior might have influenced the children’s and adolescents’ assessment of parenting style. 

It should also be noted that the SDQ is a screening instrument for identifying mental health problems, but is not a psychodiagnostic instrument that allows any conclusions about mental disorders. Furthermore, parents’ assessment using the SDQ might have been biased by the parent’s own subjective well-being. For example, parents more often rate their child’s behavior as problematic when they are exposed to high levels of stress [49]. For the mental health assessment of children and adolescents, we calculated mean values to allow comparison between the different parenting styles. However, higher mean values cannot be considered to indicate a mental disorder or a mental problem with an impact on daily life. A classification of these values into mental health disorders was not the objective of this study. Further research could address this topic and integrate the SDQ impact score. To identify debilitating mental health problems in children and adolescents, it would be useful to use standardized instruments for the assessment of clinical diagnoses in addition to the SDQ [50]. 

In terms of the moderator variables, migration status should be viewed critically, as the group of people with a migration background is heterogeneous and clear differences can be seen regarding protection and risk factors [51]. The division into no, one-sided and two-sided migration background was possibly too rough to find any differences in the association between parenting styles and adolescents’ mental health. Therefore, migration status should be considered in a more differentiated way in further studies. Further analyses could differentiate by country of origin to examine whether cultural background influences a child’s perception and sensitivity toward parenting.

Another major limitation of the present work was that the results were based on a cross-sectional analysis. Because the D-ZKE was used for the first time in the second follow-up of the KiGGS cohort study, only associations between parenting style and the mental health of children and adolescents could be analyzed. Therefore, no conclusions can be drawn about the directions of causation. However, it can be assumed that the association may run reciprocally in both directions. Longitudinal studies have found that parenting style can counteract or reinforce the development of problem behavior in children [52]. However, other studies reported that parents react with their parenting to the problem behavior of the children, and externalizing symptoms of the child can influence parenting behavior [53]. Further longitudinal studies examining the effect of parenting styles by mothers and fathers on the development of mental health in different live stages in childhood and youth are needed.

## 5. Conclusions

Parenting styles of mothers and fathers are linked to the mental health of children and adolescents. To promote a positive and sustainable influence of parenting behavior on the mental health of children, appropriate measures for prevention and health promotion are of relevance. In this context, approaches to family health promotion should focus on the different life phases and life situations of parents and children [54]. In addition, prevention and health promotion services and programs should focus on parents as they constitute the central target group impacting their children’s health development [55]. This can be achieved indirectly through different settings in which parents are involved or through institutions that deal with the health of children and adolescents and offer regional support services for parents and children. In Germany, there are several programs that aim to strengthen the parenting skills of parents for children in younger age groups (0–10 years) [55]. The German Health Inequalities Practice Database shows that few projects and programs exist that focus on promoting parenting skills among parents with older children. Therefore, it is necessary to develop further health promotion strategies. It must be considered that the life of families is influenced by various social determinants, such as SES, family structure, or migration background, all of which can impact the use of support services [55]. Therefore, there is a need for low-threshold and target-group-specific health promotion programs that make it easier for families to use these services. In summary, promoting good family cohesion and improving the parenting skills of parents may be promising strategies in promoting the mental health of children and adolescents.

## Figures and Tables

**Table 1:** Sample characteristics, by parenting style of mothers and fathers.

	Mother		Father	
	*n* (unweigh.)	% (weigh.)	*n* (unweigh.)	% (weigh.)
Total (Before Exclusion of Cases)	4596		4596	
Excluded (Missing Data)	313		595	
Excluded (Parent Did Not Live in the Same Household)	182		510	
Total (for Analysis)	4101		3491	
Parenting Styles				
Authoritative	1654	38.9	1256	34.7
Emotional Distancing	690	17.5	431	13.1
Demanding-Controlling	679	17.8	813	24.8
Permissive	1078	25.8	991	27.5
Gender				
Boys	1958	50.1	1659	50.2
Girls	2143	49.9	1832	49.8
Age Group, Years				
11–13	1784	40.9	1525	41.0
14–17	2317	59.1	1966	59.0
Socioeconomic Status				
Low	444	17.5	360	16.6
Middle	2713	63.7	2246	62.2
High	928	18.8	872	21.2
Missing	16		13	
Migration Status				
No Migration Background	3369	73.4	2857	72.7
One-Sided Migration Background	322	9.8	267	9.8
Two-Sided Migration Background	383	16.8	344	17.5
Missing	27		23	
Family Status (Living Together with Both Biological Parents in the Same Household)				
Yes	3264	78.8	3251	93.8
No	837	21.2	240	6.2
Missing	0		0	
Mother’s Status (Mother in Household)				
Biological Mother	4096	99.9	3433	99.9
Stepmother	5	0.1	5	0.1
Missing	0		53	
Father’s Status (Father in Household)				
Biological Father	3321	96.6	3362	96.7
Stepfather	129	3.4	129	3.3
Missing	651		0	
	*n* (unweigh.)	M (SD)	*n* (unweigh.)	M (SD)
SDQ Total Difficulties Score				
Total	4071	7.33 (0.11)	3468	7.08 (0.12)
Missing	30		23	

SDQ, Strengths and Difficulties Questionnaire; M, mean; SD, standard deviation.

**Table 2:** Parenting style of mothers stratified by children’s age and gender, socioeconomic status, migration status, and family status (weighted prevalence in % and 95% confidence intervals).

Mother’s Parenting Style: Boys				
	Authoritative	Emotional Distancing	Demanding-Controlling	Permissive
Total	38.5	18.3	19.2	24.1
	(35.8–41.3)	(16.2–20.5)	(16.9–21.8)	(21.9–26.4)
Age Group, Years				
11–13	51.7	11.3	21.0	16.1
	(47.3–56.0)	(8.6–14.7)	(17.6–24.8)	(13.3–19.4)
14–17	29.6	23.0	18.0	29.4
	(26.4–33.1)	(20.2–26.0)	(15.0–21.4)	(26.2–32.8)
Socioeconomic Status				
Low	41.0	21.1	25.5	12.3
	(33.8–48.7)	(15.6–28.0)	(18.4–34.3)	(8.2–18.1)
Middle	37.7	18.0	19.3	25.0
	(34.3–41.3)	(15.4–20.9)	(16.7–22.1)	(22.3–34.0)
High	37.9	16.6	12.7	32.9
	(33.4–42.6)	(13.2–20.5)	(9.3–17.0)	(28.5–37.6)
Migration Status (Migration Background)				
No	39.0	17.1	15.2	28.7
	(34.9–42.2)	(14.8–19.7)	(13.0–17.8)	(26.0–31.5)
One-Sided	39.1	16.0	27.0	18.0
	(30.8–48.0)	(9.7–25.2)	(18.7–37.2)	(12.0–26.1)
Two-Sided	35.8	24.2	29.9	10.1
	(28.1–44.3)	(18.7–30.8)	(22.3–38.7)	(6.6–15.3)
Family Status (Living Together with Both Biological Parents)				
Yes	39.1	18.4	19.5	23.0
	(36.0–42.3)	(16.0–21.1)	(16.8–22.5)	(20.7–25.5)
No	36.1	17.5	18.1	28.3
	(30.9–41.7)	(13.5–22.5)	(13.5–23.9)	(23.0–34.3)
Mother’s Parenting Style: Girls				
Total	39.2	16.8	16.3	27.6
	(36.5–42.0)	(14.8–19.0)	(14.4–18.5)	(25.3–30.1)
Age Group, Years				
11–13	48.3	14.3	14.4	23.1
	(44.0–52.6)	(11.0–18.2)	(12.0–17.2)	(19.5–27.2)
14–17	32.8	18.7	17.7	30.9
	(29.7–36.0)	(16.1–21.5)	(15.1–20.7)	(27.6–34.3)
Socioeconomic Status				
Low	34.9	23.2	20.9	21.0
	(26.5–44.4)	(17.0–30.7)	(15.0–28.4)	(14.9–28.8)
Middle	38.8	17.0	15.3	28.8
	(35.6–42.1)	(14.6–19.7)	(13.0–17.9)	(26.0–31.9)
High	42.4	11.7	14.9	31.0
	(37.4–47.6)	(8.8–15.4)	(11.7–18.9)	(26.6–35.7)
Migration Status (Migration Background)				
No	39.6	15.8	13.5	31.1
	(37.0–42.3)	(13.7–18.2)	(11.4–15.8)	(28.5–33.8)
One-Sided	34.0	23.4	18.6	24.0
	(26.5–42.4)	(17.0–31.2)	(12.9–26.1)	(16.6–33.3)
Two-Sided	39.4	18.3	28.7	13.7
	(30.8–48.7)	(12.5–26.0)	(21.7–36.8)	(8.4–21.6)
Family Status (Living Together with Both Biological Parents)				
Yes	40.1	15.2	17.0	27.7
	(36.9–43.3)	(13.1–17.6)	(14.8–19.5)	(24.2–30.3)
No	36.1	22.7	13.8	27.4
	(30.9–41.7)	(17.7–28.6)	(10.1–18.6)	(22.1–33.5)

**Table 3:** Father’s parenting style stratified by children’s age and gender, socioeconomic status, migration status, and family status (weighted prevalence in % and 95% confidence intervals).

Father’s Parenting Style: Boys				
	Authoritative	Emotional Distancing	Demanding-Controlling	Permissive
Total	33.8	12.3	27.9	26.0
	(30.9–36.8)	(10.5–14.4)	(25.2–30.7)	(23.4–28.8)
Age Group, Years				
11–13	45.9	8.2	29.1	16.7
	(41.2–50.7)	(5.9–11.3)	(24.7–34.1)	(13.5–20.5)
14–17	25.3	15.2	27.0	32.5
	(21.6–29.4)	(12.7–18.1)	(23.5–30.7)	(28.9–36.4)
Socioeconomic Status				
Low	29.2	11.6	40.5	18.8
	(20.9–39.1)	(6.8–18.9)	(32.2–49.4)	(12.3–27.5)
Middle	35.4	13.1	27.0	24.5
	(32.2–38.8)	(10.5–16.2)	(23.7–30.6)	(21.5–27.8)
High	32.9	10.8	19.6	36.8
	(28.2–37.9)	(7.9–14.6)	(14.9–25.5)	(31.3–42.6)
Migration Status (Migration Background)				
No	35.9	12.7	22.1	29.4
	(32.7–39.2)	(10.5–15.2)	(19.3–25.2)	(26.5–32.5)
One-Sided	28.7	13.3	32.3	25.6
	(21.3–37.6)	(7.3–23.2)	(23.8–42.2)	(18.3–34.7)
Two-Sided	29.6	10.4	45.6	14.4
	(21.7–39.1)	(6.2–16.9)	(38.6–52.8)	(8.9–22.3)
Family Status (Living Together with Both Biological Parents)				
Yes	34.0	12.1	27.6	26.3
	(31.0–37.1)	(10.3–14.3)	(24.8–30.5)	(23.7–29.1)
No	31.1	15.3	32.4	21.1
	(21.6–42.5)	(9.2–24.4)	(21.8–45.3)	(13.2–32.0)
Father’s Parenting Style: Girls				
Total	35.6	13.8	22.7	28.9
	(32.9–38.3)	(11.9–16.0)	(19.5–24.2)	(26.5–31.5)
Age Group, Years				
11–13	41.8	10.1	21.5	26.6
	(37.6–46.2)	(7.5–13.4)	(18.1–25.3)	(22.9–30.6)
14–17	31.2	16.4	21.9	30.5
	(27.9–34.7)	(13.8–19.4)	(19.0–25.1)	(27.1–34.2)
Socioeconomic Status				
Low	30.1	18.7	28.2	23.0
	(22.9–38.5)	(13.0–26.0)	(20.6–37.2)	(16.6–31.0)
Middle	36.5	14.2	20.0	29.3
	(33.1–40.1)	(11.8–17.0)	(17.4–22.8)	(26.1–32.8)
High	38.3	9.7	18.5	33.5
	(33.1–43.8)	(7.0–13.3)	(14.4–23.5)	(29.1–38.3)
Migration Status (Migration Background)				
No	36.7	13.7	18.4	31.1
	(33.9–39.7)	(11.5–16.3)	(16.3–20.7)	(28.2–34.2)
One-sided	36.3	13.6	23.6	26.5
	(27.1–46.6)	(8.6–20.9)	(16.4–32.8)	(18.4–36.6)
Two-sided	28.7	14.3	35.5	21.6
	(21.4–37.3)	(9.3–21.4)	(27.4–44.6)	(15.3–29.6)
Family Status (Living Together with Both Biological Parents)				
Yes	35.5	13.9	22.0	28.6
	(32.8–38.2)	(11.9–16.1)	(19.7–24.6)	(26.0–31.3)
No	36.6	12.8	17.3	33.3
	(26.0–48.6)	(7.0–22.2)	(11.4–25.4)	(24.5–43.6)

**Table 4:** Strengths and Difficulties Questionnaire total difficulties scores for girls and boys stratified by mother’s and father’s parenting style (weighted means, 95% confidence intervals, *p*-values).

	**Boys**		**Girls**		**Total**	
	**M (95% CI)**	***p*-Value**	**M (95% CI)**	***p*-Value**	**M (95% CI)**	***p*-Value**
Parenting Style: Mother						
Authoritative	7.28 (6.86–7.70)	Ref.	6.11 (5.69–6.54)	Ref.	6.69 (6.39–7.00)	Ref.
Emotional Distancing	8.50 (7.66–9.35)	**	8.70 (7.92–9.48)	***	8.60 (8.04–9.16)	***
Demanding–Controlling	9.60 (8.89–10.32)	***	8.71 (7.97–9.44)	***	9.19 (8.70–9.69)	***
Permissive	6.26 (5.72–6.80)	**	6.03 (5.55–6.51)	n.s.	6.14 (5.77–6.50)	*
Parenting Style: Father						
Authoritative	6.83 (6.36–7.30)	Ref.	5.87 (5.45–6.29)	Ref.	6.34 (6.01–6.67)	Ref.
Emotional Distancing	8.96 (7.72–10.20)	***	8.18 (7.38–8.99)	***	8.55 (7.83–9.26)	***
Demanding-Controlling	8.90 (8.18–9.62)	***	8.33 (7.65–9.00)	***	8.65 (8.13–9.17)	***
Permissive	6.07 (5.60–6.55)	*	5.75 (5.28–6.22)	n.s.	5.90 (5.55–6.26)	n.s.

* *p* < 0.05, ** *p* < 0.01, *** *p* < 0.001, n.s. not significant (*t*-test, compared with the reference group). SDQ total difficulties score range: 0 (low) to 40 (high). M, mean; CI, confidence interval; SDQ, Strengths and Difficulties Questionnaire.

**Table 5:** Multiple linear regression results for associations between Strengths and Difficulties Questionnaire total difficulties score and mother’s and father’s parenting style, adjusted for control variables (Model 1) and mediator variables (Model 2).

**Girls and Boys**	**Model 1** **(Basic Model)**			**Model 2** **(Fully Adjusted Model)**		
	**Coef.**	**95%CI**	***p*-Value**	**Coef.**	**95%CI**	***p*-Value**
Parenting style: mother						
Authoritative	Ref.			Ref.		
Emotional Distancing	2.11	(1.47–2.75)	***	1.95	(1.33–2.58)	***
Demanding–Controlling	2.61	(2.05–3.16)	***	2.43	(1.87–2.99)	***
Permissive	−0.36	(−0.87–0.14)	n.s.	−0.26	(−0.75–0.23)	n.s.
constant	7.12	(6.77–7.46)	***	7.86	(7.15–8.57)	***
Parenting Style: Father						
Authoritative	Ref.			Ref.		
Emotional Distancing	2.42	(1.67–3.16)	***	2.41	(1.69–3.13)	***
Demanding–Controlling	2.40	(1.78–3.02)	***	2.39	(1.79–2.98)	***
Permissive	−0.25	(−0.72–0.23)	n.s.	−0.07	(−0.53–0.40)	n.s.
constant	6.76	(6.37–7.16)	***	7.90	(7.22–8.58)	***

*** *p* < 0.001, n.s. not significant (*t*-test, compared with the reference group). Model 1: Adjusted for age, gender, mother’s status/father’s status (control variables). Model 2: Adjusted for control variables, SES, migration status, family status (mediator variables). Coef., β coefficient; CI, confidence interval; SES, socioeconomic status.

## Data Availability

The dataset cannot be made publicly available because informed consent from study participants did not cover public deposition of data. However, the minimal dataset underlying the findings is archived in the “Health Monitoring” Research Data Centre at the Robert Koch Institute (RKI) and can be accessed by all interested researchers on site. The “Health Monitoring” Research Data Centre is accredited by the German Data Forum according to uniform and transparent standards (http://www.ratswd.de/en/data-infrastructure/rdc, accessed on 25 June 2021). On-site access to the minimal data set is possible at the Secure Data Centre of the RKI’s “Health Monitoring” Research Data Centre, which is located at General-Pape-Straße 64 in Berlin, Germany. Requests should be submitted to Ronny Kuhnert at the Robert Koch Institute, “Health Monitoring” Research Data Centre, General-Pape-Straße 64, 12101 Berlin, Germany (email: fdz@rki.de).
